# Retraction: Case Report: Prolonged DAWS in an RLS patient under severe relational stress

**DOI:** 10.3389/fnhum.2026.1909710

**Published:** 2026-06-17

**Authors:** Frontiers Editorial Office

The journal retracts the January 2026 article cited above.

Following publication, concerns were raised regarding the scientific validity of the article and undisclosed non-financial conflicts. An investigation was conducted in accordance with Frontiers' policies and identified that the author had failed to disclose a material non-financial conflict of interest as required under Frontiers' Terms and Conditions for Users and COPE guidelines; and that the article presents significant methodological limitations and does not meet Frontiers' standards of editorial and scientific soundness for Frontiers in Human Neuroscience. Therefore, the article has been retracted.

This retraction was approved by the Chief Editors of Frontiers in Human Neuroscience and the Chief Executive Editor of Frontiers. The author does not agree to this retraction.

